# GPU based parallel acceleration for fast C-arm cone-beam CT reconstruction

**DOI:** 10.1186/s12938-018-0506-4

**Published:** 2018-06-05

**Authors:** Ken Chen, Cheng Wang, Jing Xiong, Yaoqin Xie

**Affiliations:** 1 Shenzhen Institute of Advanced Technology, Chinese Academy of Sciences, Shenzhen, China; 2 Shenzhen College of Advanced Technology, University of Chinese Academy of Sciences, Shenzhen, China

**Keywords:** Image guided therapy, Fast reconstruction, CBCT, GPU

## Abstract

**Background:**

With the introduction of Flat Panel Detector technology, cone-beam CT (CBCT) has become a novel image modality, and widely applied in clinical practices. C-arm mounted CBCT has shown extra suitability in image guided interventional surgeries. During practice, how to acquire high resolution and high quality 3D images with the real time requirement of clinical applications remain challenging.

**Methods:**

In this paper, we propose a GPU based accelerated method for fast C-arm CBCT 3D image reconstructions. A filtered back projection method is optimized and implemented with GPU parallel acceleration technique. A distributed system is designed to make full use of the image acquisition consumption to hide the reconstruction delay to further improve system performance.

**Results:**

With the acceleration both in algorithm and system design, we show that our method significantly increases system efficiency. The optimized GPU accelerated FDK algorithm improves the reconstruction efficiency. The system performance is further enhanced with the proposed system design by 26% and reconstruction delay is accelerated by 2.1 times when 90 frames of projections are used. When the number of frames used increases to 120, the numbers are 39% and 3.3 times. We also show that when the projection acquisition consumption increases, the reconstruction acceleration rate increases significantly.

## Background

With the introduction of Flat Panel Detector (FPD) technique, cone-beam computed tomography (CBCT) has become a novel image technology. FPD provides several theoretical advantages such as high space resolution, wide dynamic range, square FOV and real-time imaging capability with no geometric distortion [1]. Such good features enable CBCT to generate an entire volumetric data set in a single gantry rotation [2], and allows for verification of the delivered dose distribution [3]. The radiation dose is also reported to decrease [1, 4]. Therefore, CBCT has been widely applied in clinical applications in image guided surgery and interventional radiology, such as CBCT guidance of brachytherapy, spinal, orthopedics, thoracic and abdominal surgery [5–10]. Some groups reported that CBCT can achieve good performance in fenestrated/branched aortic endografting and small lung nodule percutaneous transthoracic needle biopsy. Some group showed that CBCT depicts considerably more small aneurysms and important anatomic details, and can be used as new gold standard in the detection of intracranial aneurysms [11, 12].

Besides, C-arm mounted CT shows especially suitable features for image guided interventions. The system is compact, therefore the patient can stay stationary during the image acquisition. Volumetric tomographic images can be combined and co-displayed with conventional 2D angiographic imaging, therefore pre-operative surgery planning, surgery device tracking and navigation, final result access and margins verification achievable [13, 14].

To acquire 3D volumetric images, several categories of algorithms are explored. One of the major category is the iterative algorithms such as ART combined with compress sensing theory using a Total Variation (TV) norm to regularize the cost function such as mentioned in [15, 16]. The main challenge of such algorithms is the cost of calculation. The time consuming process and high hardware requirement may limit their use in clinical applications. Therefore, FDK algorithm still seems to be a better choice for practical application. The filtered back projection algorithms can be further accelerated using GPU parallel techniques. From [17–19] we can see that some groups have made progress about accelerate FDK algorithm with GPU. In [18] the author reviewed how the GPU can be applied to almost every kind of image reconstruction algorithms. In [19] the author compared implementations of FDK method over different platforms to show a significant performance improvement. What is more, with a carefully designed distributed system, the algorithm can be run on high performance devices especially targeted to parallel acceleration, and the system delay can be further improved with latency hiding techniques.

In this paper, we propose a distributed system for c-arm mounted CBCT imaging system, and a GPU based acceleration method for fast CBCT reconstruction. As stated above, filtered back projection methods are more suitable for real time clinical 3D imaging acquisition than iterative optimization kind methods, and GPU parallel acceleration can be applied. Although the GPU parallel acceleration technique is not new, the acceleration plan can be further optimized with geometric symmetry and a proper system design. Therefore we propose to further optimize the FDK algorithm based on geometric symmetry, and implement it with GPU parallel acceleration techniques. We also propose to design a delay hiding scheme based on a distributed system layout connected via TCP/IP protocol, making full use of the projection acquisition consumption to hide the reconstruction delay. The rest of the paper is organized as follows: in “Methods” section we explain the details of system design, the GPU accelerated FDK algorithm implementation and latency hiding scheme. In “Experiment results and discussion” section, we show the reconstruction result and evaluate the system performance.

## Methods

### System design

To achieve a better acceleration effect a high performance GPU specially designed for computing task may be required, which may make ordinary hardware system not suitable. Besides, as the main constrain of the system efficiency, the reconstruction process is relatively an independent part of the image chain, which implies further change or update will not intervene with other parts of the system. Therefore, a pluggable computing unit with a distributed system architecture is favored. We briefly describe our system design as follows.

**Fig. 1:**
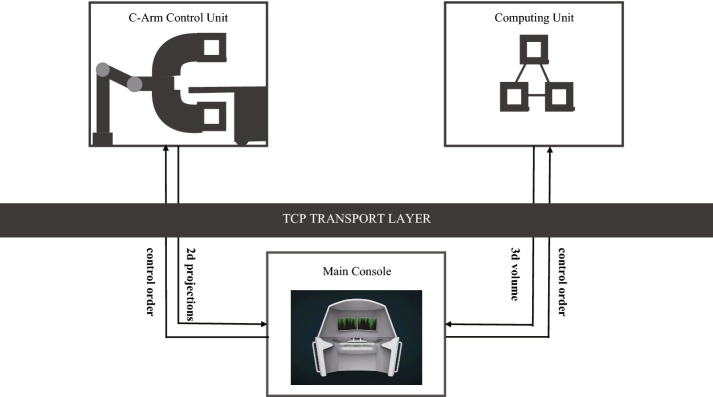
System architecture. The system is divided into three parts: a C-arm control unit, a computing unit, and a main console. The units are connected through TCP/IP protocol for data and order transmission

The system is composed of three main parts as shown in Fig. 1. The C-arm control unit controls the gantry rotation, acquires projection images. The computing unit reconstructs 3D volume data from 2d projection images. The main console unit controls the image chain, sending orders and requests to corresponding units, manages data stream and display system statues and 2D/3D visualization. The three parts are connected and communicating via TCP/IP protocol to transmit data and orders.

### GPU accelerated FDK algorithm

**Fig. 2:**
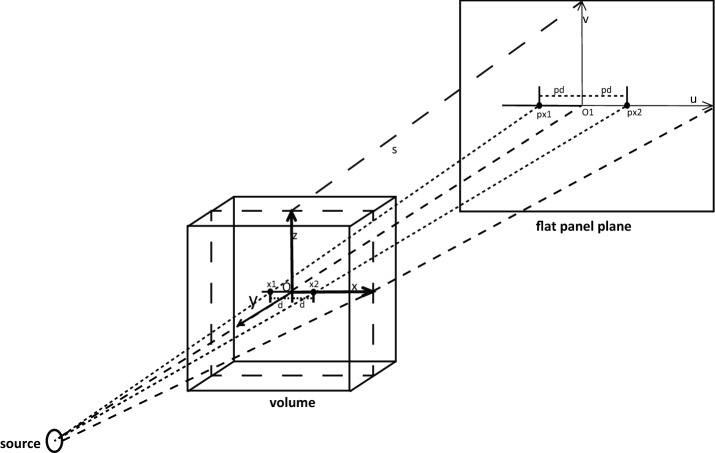
FDK reconstruction coordinate system definition. The definition of the volume voxel coordinate system axis X, Y, Z with origin O and the flat panel plane coordinate system U,V with origin O1. Assume voxel x1 and x2 are symmetric with the plane s formed by axis Z and axis V, and the distance to plane s is d, their projections on the flat panel plane px1 and px2 are symmetric with axis V, with the same distance pd to axis V. Therefore we only need to calculate one half of the volume voxels. The calculation for the other half can be accomplished by a mirror action

FDK algorithm was originally proposed by Feldkamp et al. [20] for approximate 3D filtered back projection reconstruction with circular trajectory and flat panel detectors. The algorithm can be briefly represented as follows:

1 $$\begin{aligned} f(x,y,z) = \frac{1}{2}\int \limits _0^{2\pi } {\frac{{{DSD^2}}}{{{U^2}}}} \left\{ {\left( {{\phi _\theta }(u,v)\frac{{DSD}}{{\sqrt{DS{D^2} + {u^2} + {v^2}} }}} \right) * h(u)} \right\} d\theta \end{aligned}$$

where

2 $$\begin{aligned} U = DSD - z\sin (\theta ) + y\cos (\theta ) \end{aligned}$$

and we define the two weighting factors as

3 $$\begin{aligned} {W_1}= & {} \frac{{{DSD^2}}}{{{U^2}}}\nonumber \\ {W_2}= & {} \frac{{DSD}}{{\sqrt{DS{D^2} + {u^2} + {v^2}} }}, \end{aligned}$$

while ${DSD}$ is the distance from source to the detector, ${{\phi _\theta }}$ is the projection data, h is the filter kernel, $\mathrm{W}_1$ and $\mathrm{W}_2$ are weighting factors to compensate the different ray length. u, v are the projection of the ray with angle $\theta$ on the flat panel detector. The coordinate system is defined in Fig. 2. O is the center of FOV as well as the C-arm rotation geometry, O1 is the projection of O on flat panel detector, and is defined as the origin of projection images.

The nature of FDK algorithm is especially suitable for parallel acceleration. The main idea of GPU parallel acceleration technique is that the GPU provides far more arithmetic units than general purpose processors, and a stream processing scheme for high efficient parallel computing. For each element of an input stream data, a kernel is defined to carry out arbitrary calculations to produce an output stream data. Therefore GPU acceleration is especially suitable for pixel-wise operations, turning iterative loops of similar operations into parallel execution.

**Fig. 3:**
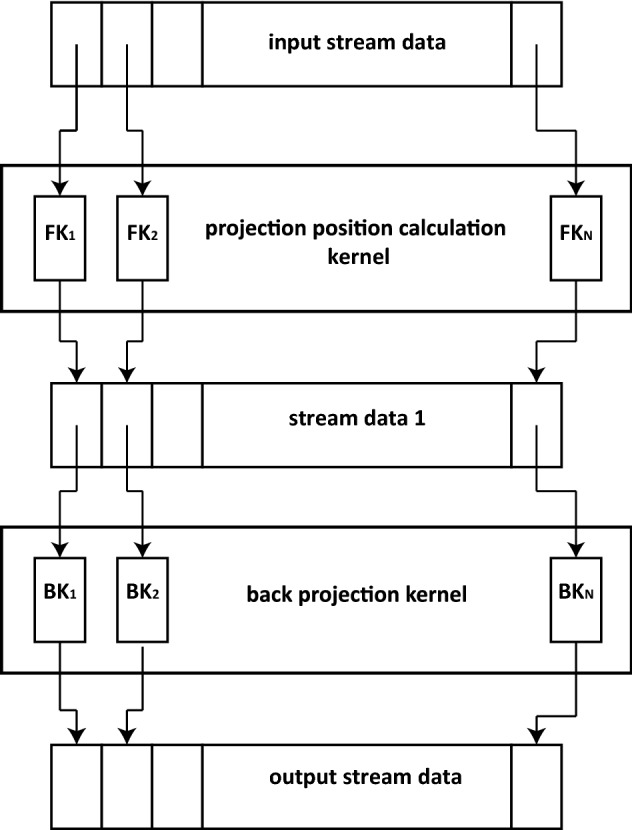
Stream processing scheme for FDK algorithm. Each volume voxel is considered as an element of the input data stream. The projection position calculation kernel provides each element a parallel thread to calculate the corresponding projections on the flat panel plane. The results form a new data stream as the input to the back projection kernel, which performs the back projection and calculate the value of the corresponding volume voxel parallelly

For FDK algorithm, the projection position calculation process to determine the projection position of each volume voxel on the flat panel detector plane, and the calculation of the weighting factor ${W_1}$ and ${W_2}$ for each volume voxel in the back projection procedure are most time consuming. However, these calculations are highly similar for each volume voxel, and there is no dependency between each voxel, so intuitively the voxel-wise iterative loop can be parallelized by assigning a kernel to each volume voxel to improve efficiency. We also observe that ${W_2}$ is only dependent on the projection coordinate on the detector plane, therefore the calculation of ${W_2}$ can be separated from the voxel wise calculation and treated as a filtering process before the back projection process. The stream processing scheme of the reconstruction for an arbitrary frame can be briefly described as Fig. 3.

With the geometric symmetry, the number of kernels needed can be further optimized. For a pair of voxels symmetric with a plane s formed with z axis and v axis in Fig. 2, the projection positions on the flat panel detector plane are also symmetric with axis v, and the two weighting factors are the same. Therefore, we only need calculations for one half of the voxels along axis x+ or x−, and the calculations for the other half can be achieved with a mirror action. For the horizontal filtering, convolution can be achieved efficiently by fast Fourier transform method CUDA provides.

### Latency hiding implementation

**Fig. 4:**
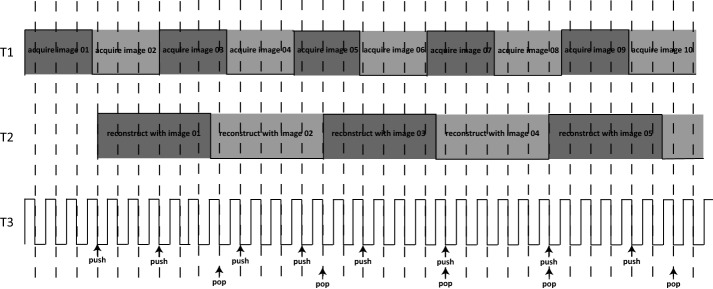
System control time sequences. At every falling edge of T3, the states of T1 and T2 are checked to decide a push or pop action on the projection queue. When the reconstruction of an arbitrary frame is in progress, the acquisitions for the next frames can be carried out at the same time to form a latency hiding scheme. The push and pop action controls the frame queue for reconstruction

With the distributed system design, the efficiency of reconstruction process can be further boosted with a latency hiding technique. As stated in the last section, the only dependency of the reconstruction with an arbitrary projection frame is the acquisition of the frame, while the projection acquisition does not depend on the reconstruction result. Therefore the efficiency can be further improved on system level by designing a parallel control time sequence of the image chain to make full use of system time consumption such as C-arm rotation, image acquisition and processing, and data transmission, etc., which we define as the projection acquisition consumption. The control is designed as follows:

We design three control time sequences, T1 T2 and T3, all of which take values of − 1 and + 1, as shown in Fig. 4. T1 represent the statue of projection image acquisition process, when the C-arm has moved to an arbitrary position and the image is acquired and transmitted to computing unit via TCP/IP, T1 is set opposite. T2 represents the statue of reconstruction process. When an arbitrary frame is filtered back projected to the volume data, the T2 value is set opposite. T3 is the control signal to synchronize T1 and T2. T3 is generated by a timer with a very small time interval. Whenever T3 has a falling edge, T1 and T2 signal is checked. When T1 has a falling/rising edge, the newly acquired image and corresponding parameters are pushed into a queue L. When T2 has a falling/rising edge, the first image and the parameters are popped out of the queue, and corresponding memory is released. When the queue L is empty, the reconstruction is complete, and all the time sequences and the queue are reset. Compared with the linear image chain system, the control sequences allow the usually less time consuming image acquisition process be carried out as the reconstruction progressing. In the experiment section we show that the latency hiding scheme can further improve system performance.

### Experiment design

We test our proposed method from two aspects. First we show the reconstruction result of our methods. We use a Shepp–Logan numeric phantom for quantitative evaluation of reconstruction accuracy. We also show reconstruction results for phantoms of blood vessel, head and foot respectively. Then we discuss the efficiency of our proposed method. We first discuss solely the reconstruction process by comparing our proposed method with other methods either with different methodology, or with different acceleration technique. We then evaluate the system performance enhancement by introducing two acceleration ratio. The first ratio, system performance ratio ${\beta _{sys}}$ represents the system performance boost by comparing the system overall delay of our proposed system and a linear image chain system, yielding ${\beta _{sys}} = 1 - {T_{prop}} / (T_{recon} + T_{acq})$, where ${{T_{prop}}}$ is the average of the measured system delay of our proposed system, and ${{T_{acq}}}$ and ${{T_{recon}}}$ are the average time consumption for projection acquisition and reconstruction process respectively. Another ratio, reconstruction acceleration ratio ${\beta _{recon}}$ aims to evaluate the reconstruction efficiency improvement provided by our proposed system, yielding $\beta _{recon} = T_{recon} / (T_{prop} - T_{acq})$. The average is acquired over a test data set of 10 gantry rotations of our C-arm mounted CBCT.

### System and environment setup

We test our method on our designed C-arm imaging system. The C-arm DSD is 1000 mm, SAD is 500 mm. The X-ray source is imd X-RAY TUBE HEAD E-40R, the parameter is 65 kv 2 mA with an exposure time of 15 ms. The projection image has a dimension of $1560 \times 1440$ pixels, with a $0.18 \times 0.18$-mm resolution, with the acquisition angle averagely covers a range of 210°. A Quadro 6000 is used for GPU acceleration, with 256 threads in parallel.

## Experiment results and discussion

### 3D reconstruction evaluation

We first discuss the 3D reconstruction result from our system, to show that our method does not compromise the reconstruction accuracy. We test our method on a numeric phantom for quantitative analysis by evaluating the reconstruction error with the ground truth. We also show reconstruction result of a blood vessel phantom, a head phantom and a foot phantom respectively, to show that our proposed method is capable of correctly reconstructing the interested structure from actual projection data acquired from a clinical practical C-arm CBCT.

**Fig. 5:**
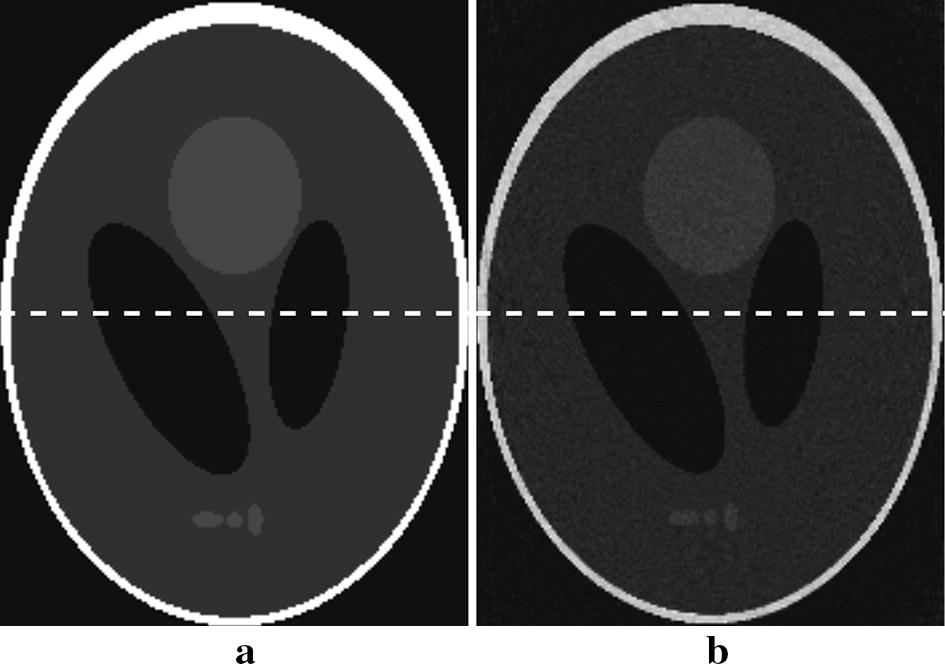
Reconstruction result of a standard Shepp–Logan numeric phantom. **a** Standard Shepp–Logan phantom; **b** reconstruction result

**Fig. 6:**
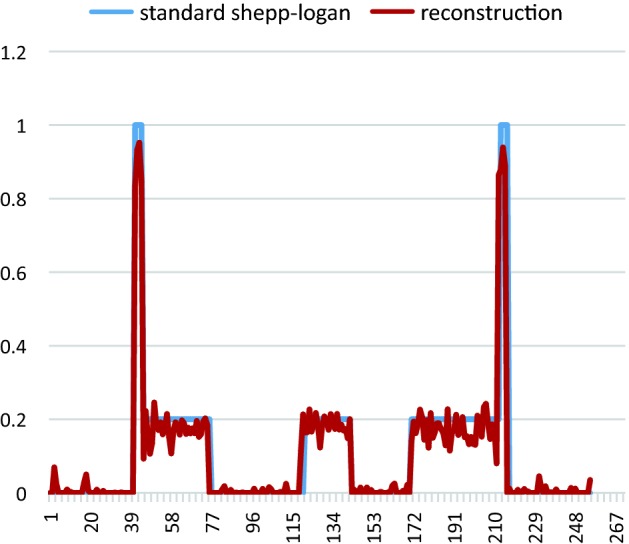
Line profile difference between reconstruction and standard phantom along the dash line shown in Fig. 4. The black line represents the standard Shepp–Logan phantom; the red line represents reconstruction result. Along the line the relative error is 2%

**Fig. 7:**
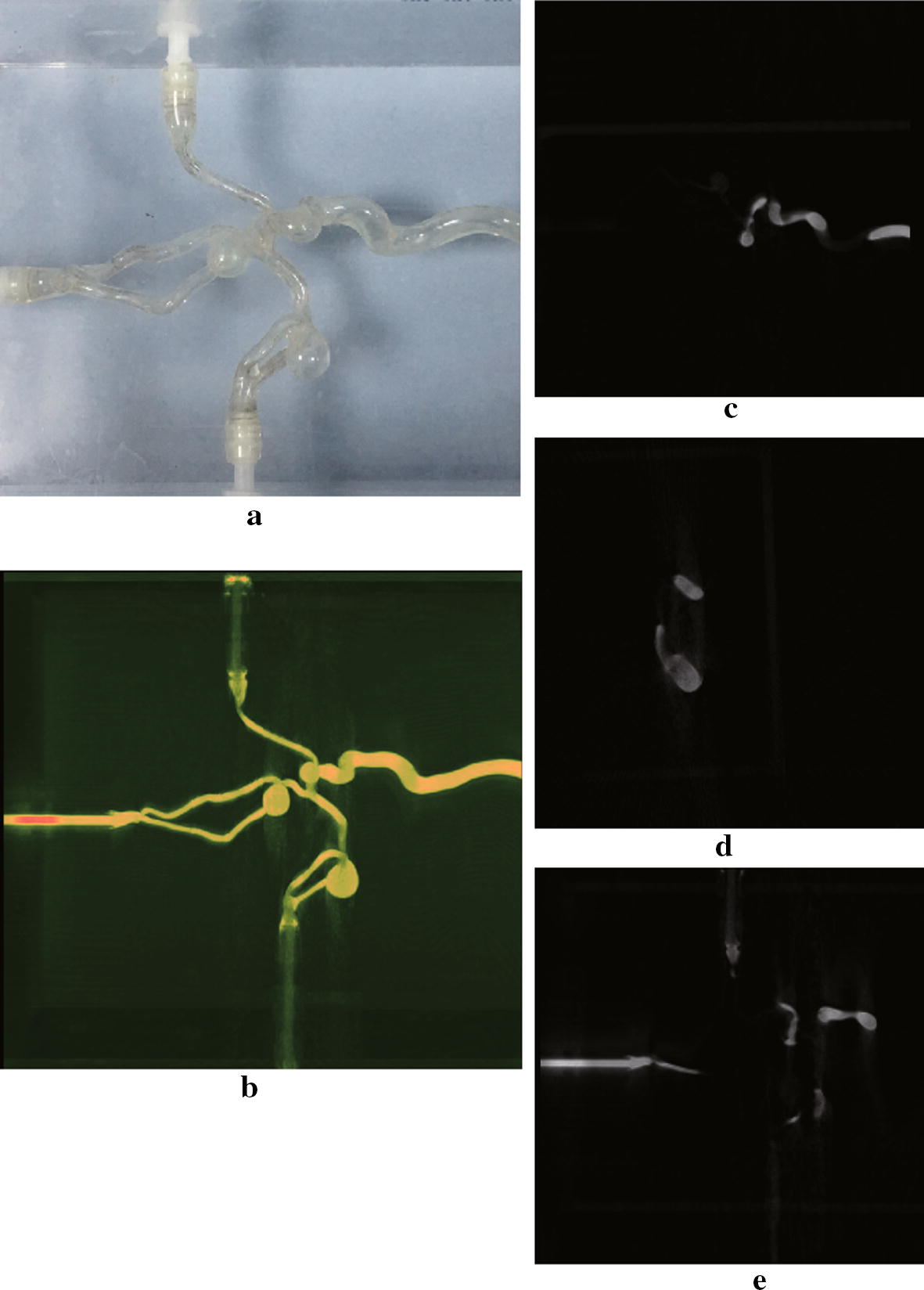
Reconstruction result of a vessel phantom. **a** The brain blood vessel phantom with 3 cranial aneurysms. **b** MIP of reconstruction result. **c**–**e** Sagittal view, coronal view and transverse view

**Fig. 8:**
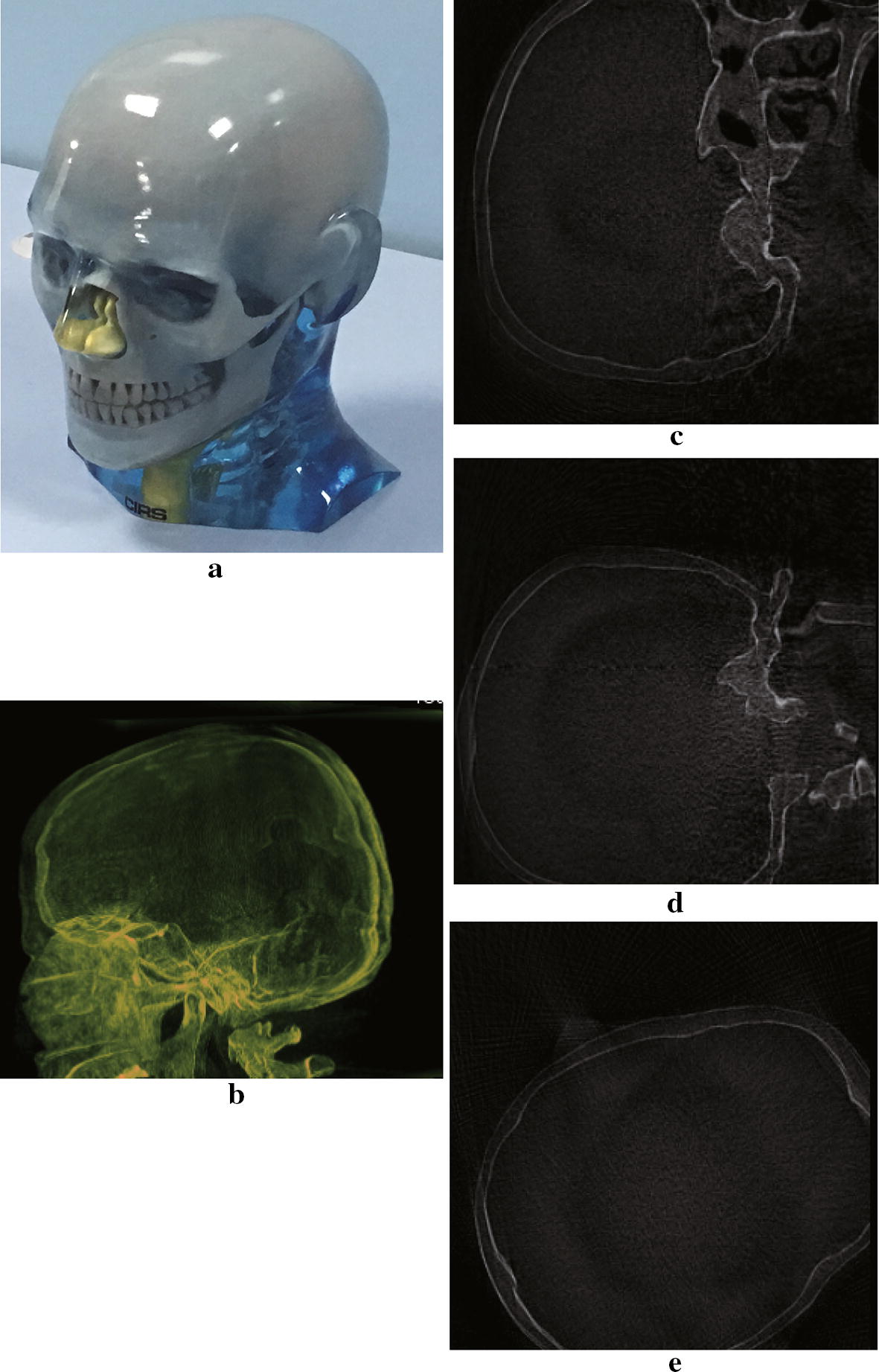
Reconstruction result of a head phantom. **a** The head phantom. **b** MIP of reconstruction result. **c**–**e** Sagittal view, coronal view and transverse view

**Fig. 9:**
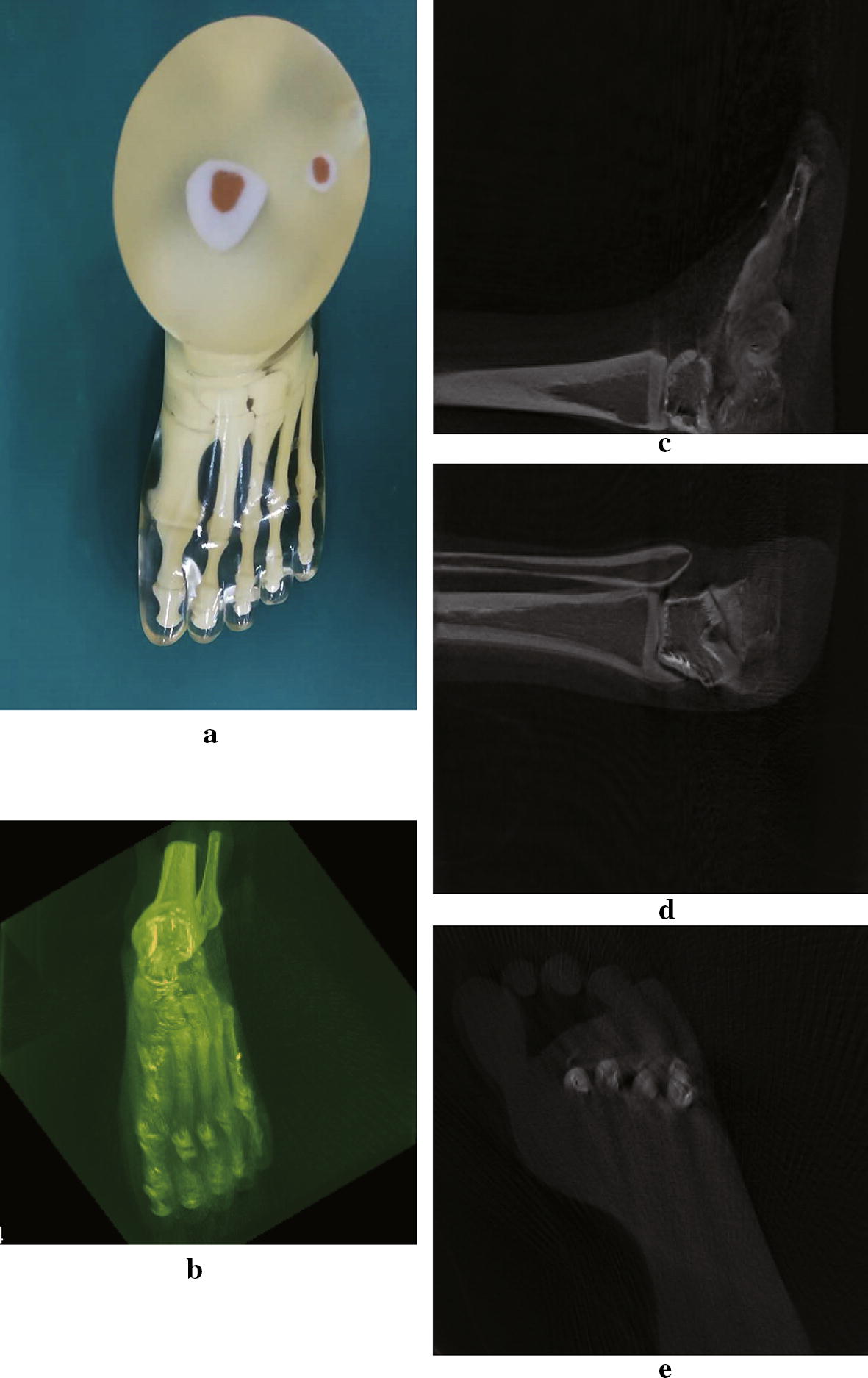
Reconstruction result of a foot phantom. **a** The foot phantom. **b** MIP of reconstruction result. **c**–**e** Sagittal view, coronal view and transverse view

The Shepp–Logan numeric phantom we use to test our method is shown in Fig. 5. We compare the reconstruction result and the ground truth on line profiles shown in Fig. 5, and the result is shown in Fig. 6. If we define the relative error between reconstruction ${f_{rec}}$ and the ground truth ${f_0}$ as $I = \frac{{{\Sigma ^N}(abs({f_{rec}} - {f_0})/{f_0})\,*\,100\% }}{N}$, where N is the number of pixels counted, the relative error along the profile line is 2%, which suggests the reconstruction basically preserves the feature of the phantom. In Fig 7 we show the reconstruction of a Elatras brain blood vessel phantom with 3 aneurysms. The head and foot phantom and the reconstruction results are shown in Figs. 8 and 9 respectively.

### Efficiency analysis

In Table 1, we present the time consumption ${{T_{recon}}}$ of proposed GPU accelerated FDK, non-accelerated FDK, IPP accelerated FDK and an iterative TV norm regulated algebra reconstruction technique (TV-ART) algorithm, with different number of projection frames used for reconstruction. Theoretically the acceleration ratio should be equal to the number of parallel threads, which in our case should be 256. However, due to host–device communication and data transmission delay, only an acceleration ratio of 69 is achieved. Nevertheless, from Table 1 we can still show that GPU acceleration can significantly improve calculation efficiency, and also achieve a better performance enhance than existing acceleration techniques such as IPP, even when tested on a relatively low performance GPU.

**Table 1:** Comparison of GPU, IPP accelerated FDK and GPU accelerated TV-ART algorithms time consumption ${{T_{recon}}}$

	Non-accelerated FDK	IPP FDK	GPUFDK		GPU ART
Frames	90	90	90	120	120
Time	964 s	53.40 s	14.28 s	25.76 s	32 min

In Table 2, we present the total system delay of our proposed image chain ${{T_{prop}}}$, and the time consumption ${{T_{acq}}}$ solely for projection acquisition. Combining Tables 1 and 2, we calculate the linear image chain system delay ${T_{linear}} = {T_{acq}} + {T_{recon}}$ and the reconstruction delay of our proposed system defined as ${T_{recon\_prop}} = {T_{prop}} - {T_{acq}}$, and a summary is provided in Table 3. We compare the system delay of our proposed system ${{T_{prop}}}$ from Table 2 with the linear image chain system delay ${T_{linear}}$ from Table 3 to show the system performance improvement under different circumstances. We also compare the reconstruction delay of our proposed system ${T_{recon\_prop}}$ from Table 3 with the reconstruction time ${{T_{recon}}}$ from Table 1 to show the reconstruction efficiency boost. The two acceleration ratios ${\beta _{sys}}$ and ${\beta _{recon}}$ are also calculated. We can see that when we use 90 frames of projection data, a linear image chain system delay ${{T_{linear}}}$ is 29 s, while our proposed method yields a system delay of 21.49 s, which implies a ${\beta _{sys}}$ of 26%. The reconstruction delay ${{T_{recon\_prop}}}$ is 6.78 s, which yields a ${\beta _{recon}}$ of 2.1. When we use 120 frames in case 1, the numbers are 46.29, 28.17 and 7.64 s respectively, yielding a ${\beta _{sys}}$ of 39% and a ${\beta _{recon}}$ of 3.3. We can infer that, as the frames of 2d projections used for 3D reconstruction increases, the system performance benefit more from our proposed method.

**Table 2:** Summary of system delay ${{T_{prop}}}$ and projection acquisition cost ${{T_{acq}}}$

	System delay			Projection acquisition cost		
Frames	90	120 case 1	120 case 2	90	120 case 1	120 case 2
Time (s)	21.49	28.17	50.53	14.72	20.53	50.14

**Table 3:** Summary of linear system delay ${{T_{linear}}}$, proposed reconstruction delay ${{T_{recon\_prop}}}$, system performance ratio ${\beta _{sys}}$ and reconstruction acceleration ratio ${\beta _{recon}}$

	Linear system delay (s)	Proposed reconstruction delay (s)	System performance ratio (%)	Reconstruction acceleration ratio
90 frames	29.00	6.78	26	2.1
120 frames LAN	46.29	7.64	39	3.3
120 frame WAN	75.90	0.39	33	66

**Fig. 10:**
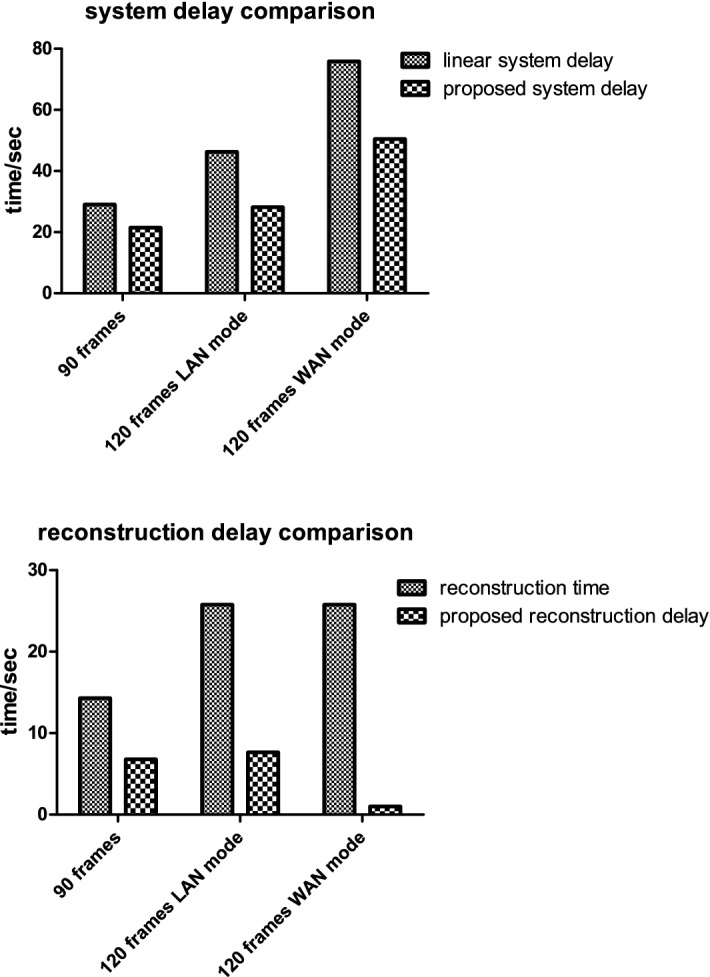
Efficiency comparison. We compare the linear image chain system with our proposed system design under different circumstance. (90 frames, 120 frames with LAN connection, 120 frames with WAN connection) up: the system delay comparison. We can conclude that our proposed design further improves the system performance. Down: the reconstruction delay comparison. We can conclude that the latency hide design significantly reduces reconstruction delay, and when other part of the image chain is dominant, the reconstruction process can be almost fully hidden, yielding nearly no reconstruction delay

We also simulate a remote connection situation in case 2 by connecting the system units through WAN while in case 1 the system units are connected via LAN with the same router. Although the system performance enhancement ratio ${\beta _{sys}}$ is 33.4%, the reconstruction delay decreases to 0.39 s, which is accelerated by ${\beta _{recon}}$ of 66 times. With the reconstruction optimization techniques, when we significantly increase the reconstruction efficiency and the projection acquisition consumption is dominant, the reconstruction delay can be very low. When the time consumption for reconstruction and project acquisition equals, the system performance benefit is maximized, and the reconstruction process can almost be hidden. The upper limit of the system performance enhancement ${\beta _{sys}}$, which can be approached but not achievable, is 50%. A more straight forward comparison is shown in Fig. 10.

## Conclusion

In this paper, we propose a GPU parallel acceleration based fast CBCT 3D reconstruction method. We describe how the FDK algorithm is parallelized, and also a control time sequence designed to further improve efficiency by hiding system latency. We can see that our proposed method significantly improves system performance. GPU parallel acceleration significantly improves the FDK reconstruction process. Our designed latency hiding scheme further improves the system performance. When 90 frames of projections are used for reconstruction, our proposed method improve system delay by 26% and the reconstruction delay by 2.1 times. When 120 frames are used, the numbers are 39% and 3.3 times. We also show that when the projection acquisition delay is dominant in the image chain, the reconstruction process can be almost fully hidden, yielding a significant improvement of reconstruction delay, which is 66 times in our case.

Although the quality of the reconstruction volume may suffer from the approximate nature of the filtered back projection algorithms compared with iterative algorithms such as ART, we show that the features of interest are acceptably preserved. A typical ART kind algorithm as described in [16] may take more than 40 min for reconstruction, while our proposed method only take 20+ seconds under the same circumstance. To trade off between image quality and real time requirement, our proposed method will be more suitable for clinical practice. A distributed system design with TCP/IP protocol makes the system pluggable and adaptive. With this design, algorithm and hardware update of reconstruction techniques can be fulfilled more easily, the system is also prepared for further expansion, such as multi-task support and distant network medical applications.
